# 

**Published:** 1897-12

**Authors:** 


					﻿Vol. LI.	DECEMBER, 1897.	No. 12.
THE
DENTAL REGISTER
A MONTHLY JOURNAL.
Devoted to the Interests of the Profession.
EDITED BY
J. TAFT, D. D.S.
ASSOCIATE EDITORS :
WILL. H. WHITSLAR,D. D. S.	N. S. HOFF,D.D.S.
PUBLISHED BY
SAM’L A. CROCKER & CO.,
OHIO DENTAL AND SURGICAL DEPOT,
35, 37 and 39 West Fifth Street,	CINCINNATI, OHIO.
Entered at the Pustoffice at Cincinnati, 0., as second class mail matter.
TERMS: $2.00 per Annum in Advance. Single Copies, 25 Cts.
				

